# Translation and cultural adaptation of the Shame and Stigma Scale (SSS) into Portuguese (Brazil) to evaluate patients with head and neck cancer^☆^

**DOI:** 10.1016/j.bjorl.2016.10.005

**Published:** 2016-11-09

**Authors:** William Eduardo Pirola, Bianca Sakamoto Ribeiro Paiva, Eliane Marçon Barroso, David W. Kissane, Claudia Valéria Maseti Pimenta Serrano, Carlos Eduardo Paiva

**Affiliations:** a Hospital de Câncer de Barretos, Programa de Pós-graduação Stricto Sensu em Oncologia, Barretos, SP, Brazil; b Hospital de Câncer de Barretos, Grupo de Pesquisa em Cuidados Paliativos e Qualidade de Vida Relacionada à Saúde (GPQual), Barretos, SP, Brazil; c Monash University, Departments of Psychiatry and Palliative Care, School of Clinical Sciences at Monash Health, Victoria, Australia; d Faculdade de Ciência e Letras de Bebedouro, Graduação em Letras, Bebedouro, SP, Brazil; e Hospital de Câncer de Barretos, Departamento de Oncologia Clínica, Divisão Mama e Ginecologia, Barretos, SP, Brazil

**Keywords:** Quality of life, Shame, Social stigma, Head and neck cancer, Qualidade de vida, Vergonha, Estigma social, Neoplasias de cabeça e pescoço

## Abstract

**Introduction:**

Head and neck cancer is the sixth leading cause of death from cancer worldwide and its treatment may involve surgery, chemotherapy and/or radiation therapy. The surgical procedure may cause mutilating sequelae, that can alter patient self-image. Thus, head and neck cancer is often connected to the negative stigma with decreased quality of life. Few studies assess the social stigma and shame perceived by patients with head and neck cancer.

**Objective:**

To perform the translation and cultural adaptation of the Shame and Stigma Scale (SSS) into Portuguese (Brazil).

**Methods:**

Two independent translations (English into Portuguese) were carried out by two professionals fluent in the English language. After the synthesis of the translations, two independent back-translations (from Portuguese into English) were performed by two translators whose native language is English. All translations were critically assessed by a committee of experts consisting of five members. A sample of 15 patients answered the Brazilian Portuguese version of the SSS to carry out the pretest. At this step, the patients were able to suggest modifications and evaluate the understanding of the items.

**Results:**

There was no need to change the scale after this step. Based on the previous steps, we obtained the Portuguese (Brazil) version of the SSS, which was called “Escala de Vergonha e Estigma”.

**Conclusion:**

The Portuguese (Brazil) version of the SSP was shown to be adequate to be applied to the population with HNC and, therefore, the psychometric properties of the tool will be evaluated during following steps.

## Resumo

**Introdução:**

O câncer de cabeça e pescoço corresponde à sexta causa de morte por câncer no mundo; seu tratamento pode envolver cirurgia, quimio e/ou radioterapia, sendo que a cirurgia pode acarretar sequelas mutiladoras, podendo alterar a autoimagem do paciente. Assim, o câncer de cabeça e pescoço é muitas vezes ligado ao estigma negativo, com diminuição da qualidade de vida. Poucos instrumentos avaliam o estigma social e a vergonha percebidos por pacientes com câncer de cabeça e pescoço.

**Objetivo:**

Traduzir e adaptar culturalmente a *Shame and Stigma Scale* (SSS) para o português/Brasil.

**Método:**

Realizaram-se duas traduções independentes (do inglês para o português) por dois profissionais fluentes na língua inglesa. Após a síntese das traduções, duas retrotraduções independentes (do português para o inglês) foram realizadas por dois tradutores cuja língua materna é o inglês. Todas as traduções foram analisadas criticamente por um comitê de especialistas composto por cinco membros. Uma amostra de 15 pacientes respondeu à versão em português/Brasil da SSS para realização do pré-teste. Nesta etapa, os pacientes puderam sugerir modificações e avaliar o entendimento dos itens.

**Resultados:**

Não houve necessidade de alteração da escala após esta fase. A partir das etapas anteriores, obteve-se a versão em português/Brasil da SSS, que foi denominada de Escala de Vergonha e Estigma.

**Conclusão:**

A versão em português/Brasil da SSS se mostrou apta para ser aplicada na população com câncer de cabeça e pescoço; portanto, as propriedades psicométricas do instrumento serão avaliadas na etapa seguinte.

## Introduction

Head and Neck Cancer (HNC) corresponds to neoplasms in the anatomical-topographic region of the upper aerodigestive tract; among them, approximately 90% are squamous cell carcinoma (SCC).^1^ Of the 580,000 new cancer cases estimated in Brazil in 2016, more than 15,000 cases are located in the oral cavity, whereas more than 7000 new cases are located in the larynx. Thus, considering these two locations together, HNC occupies the second and ninth places in terms of incidence among men and women, respectively.^2^

The main risk factors associated with HNC are smoking and alcohol intake. Among other factors, the Human Papillomavirus (HPV) is directly related to oropharyngeal tumors,^3^ while the Epstein–Barr virus is associated with nasopharyngeal neoplasms.^4^ The incidence rate of these neoplasms has declined in countries where smoking also decreased.^4^ However, 5–30% of patients diagnosed with HNC never smoked.^5^

HNC is an aggressive, debilitating condition, associated with pain and weight loss. The treatment can be carried out by surgical and/or radiation therapy, systemic treatment with chemotherapy and/or new chemotherapeutic drugs. To define the type of treatment, it is mandatory to assess the tumor biological characteristics and perform a correct clinical staging. Moreover, it is important to assess the patient's clinical condition and critically discuss with patients the complications that treatment may entail. Whenever possible, the patient should actively participate in shared decisions on the decision-making of their treatment.^6^

The adverse events caused by chemotherapy and radiotherapy, in addition to the potential mutilations resulting from surgery, may negatively impact the quality of life reported by patients.^7^

The surgical procedures can cause alterations in patient body image, whether due to changes in the skin or the resulting scars.^8^ However, when patients require some type of surgical intervention on the face, the self-image impact changes significantly, as the face is the main body part that makes eye contact with people. Physical changes cause patients to define their body image through new experiences, changing their feelings and attitudes. Individuals who suffered facial mutilations have traumatic experiences that may cause a negative impact on quality of life and decrease self-esteem.^8^

Studies have shown a significant decrease in the quality of life of cancer patients after treatment, especially regarding socialization with others outside their usual contacts, or within their own families. Such social interactions can be affected by several factors, such as depression, shame due to voice alterations, or shame due to one's physical appearance.^9^, ^10^

HNC and its treatment sequelae are mainly associated with the negative stigmata related to deterioration, death and suffering. Thus, both patients and their families go through a readaptation process, mainly regarding body image.^11^ The stigma feeling can be defined as a sense of shame, the result of a large negative impact on physical appearance, such as mutilation caused by surgical procedures. Patients with these symptoms may experience depression and social isolation.^12^

Therefore, it becomes necessary to objectively identify how interventions related to treatment and the disease itself may interfere with the life of patients with HNC. Through a literature review, we found a tool that objectively evaluates the sense of shame and stigma reported by the patient. The Shame and Stigma Scale (SSS) consists of 20 items, divided into four domains. Eight items are related to shame and appearance, three are related to social isolation, six to the feeling of stigma and three items to regret. Responses are graded on a Likert scale ranging from 0 to 4, where 0 corresponds to “never”; 1 to “seldom”; 2 to “sometimes”; 3 to “often” and 4 to “all the time”. The SSS has been originally developed in English and demonstrated adequate psychometric properties in the original study.^12^

The aim of this study was to perform the translation and cultural adaptation of the SSS scale into the Portuguese language spoken in Brazil.

## Methods

### Study design

This study is part of a larger project, of which the objective is to validate the SSS to be applied in Brazil. In this article, we will describe in detail the translation and adaptation steps, whereas the evaluation of the psychometric properties is still undergoing. This study is considered a methodological study, aimed to validate a health care assessment tool.

### Ethical aspects

This study was approved by the Research Ethics Committee of the hospital where the study was carried out (Opinion N. 914/2015). All participants were invited to participate in the study and signed the free and informed consent form. Patients with functional and/or esthetic sequelae from the treatment of head and neck tumors, aged 18 years and older, who were aware of the cancer diagnosis were invited to participate in the study. Patients with neuropsychiatric disorders that hindered the interpretation of the scales used in the study, according to the investigator's assessment, were excluded.

Patients were recruited by convenience from the Dentistry Department of an Oncological Hospital. All interviews were carried out by a single interviewer in a private room, where only the patient and the interviewer were present, to maintain the confidentiality of responses and decrease the patient's inhibition when answering the questionnaire. The interviewer did not participate in the patients’ treatment, being responsible only for data collection. Fifteen patients were invited to participate in the study and there were no refusals.

### Translation and cultural adaptation process

The SSS was submitted to the translation and cultural adaptation process according to Beaton et al.^13^ The translation process takes into account the performance of five phases, which comprise translation, synthesis of translations, back-translation, committee of experts and pretest.

– Translation: performed by at least two translators, whose native language is the one into which the tool will be translated, and who are fluent in the original language of the tool.
– Synthesis of translations: carried out in partnership with the translators and researchers responsible for the study. The aim of this step is to finalize a new version of the tool in the language proposed for the study.
– Back translation: reverse translation by translators fluent in the language into which the tool is being translated and in the language of the original tool. For this step, the translators should not have prior knowledge of the original tool, so that the translation process is not induced. In this study, the back-translated versions were compared to the original tool through collaboration with one of the authors who originally developed the scale.
– Committee of experts: it analyzes all versions according to semantic-idiomatic, conceptual and cultural parameters of the language, and further consolidates a pre-final version to be applied on a smaller sample of patients. It has the autonomy to modify, maintain or delete items that are ambiguous or irrelevant. In this study, the committee members used scores for all versions of the scale, with scores ranging from 1 (not representative item) to 4 (representative item). For all items of the scale and their respective answers, three categories were assessed: semantic/idiomatic, conceptual and cultural.^14^ Based on these assessments, we calculated the Content Validity Index (CVI) considering the answers with scores 3 and 4. Thus, the CVI of each item corresponds to the sum of the number of responses 3 and 4, divided by the total number of responses. For the evaluated item to be considered appropriate, a minimum value of 0.80 was accepted.^15^
– Pretest: at this phase, patients were invited to participate and answered the pre-final version of the tool. All of them were asked about possible suggestions that could improve the scale understanding. Additionally, the researcher responsible for applying the pre-test objectively assessed, according to his opinion, whether the patients appropriately understand the items and answers. Patients were also asked if any of the items seemed embarrassing to them.

**Figure 1:**
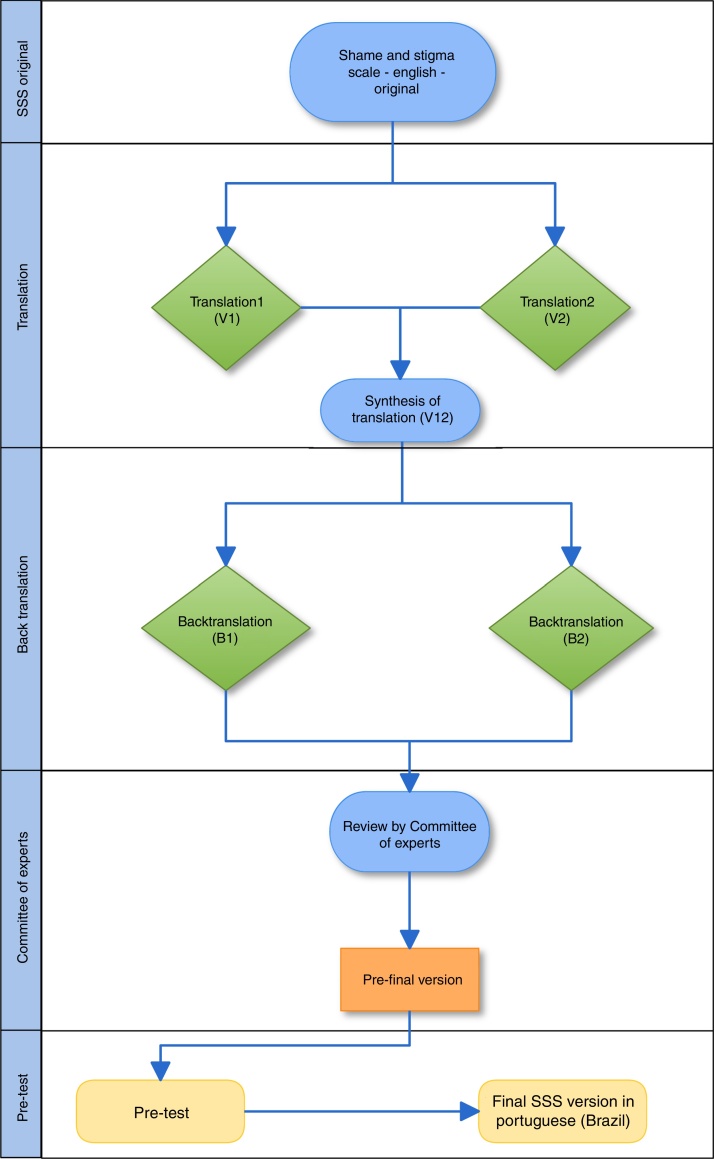
Flowchart of the translation and cultural adaptation process.

Fig. 1 shows all phases of the translation and cultural adaptation process.

## Results

### Translation and cultural adaptation process

Two bilingual translators (Portuguese-English), natives of Brazil, were invited to translate the SSS version for the translation process from the English language into Portuguese (Brazil); the new versions of the scale were called V1 and V2. None of the translators had prior knowledge of the scale. Both versions in Portuguese were analyzed by a group consisting of three authors of this study, which aimed to synthesize the translated versions into a new version (V12).

Subsequently, the result of the first version of the scale in Portuguese (Brazil) was submitted to back-translation into English by two native English speakers, an American and an Australian translator; both were fluent in Portuguese (these versions were called B1 and B2).

In this study, the Committee of Experts consisted of five members: (1) Clinical Oncologist, with a Post-doctoral degree in Oncology, with more than 10 years of clinical experience in oncology, with prior experience in the validation of assessment tools; (2) Nurse, with a Post-doctoral degree in Oncology, with more 15 years of clinical experience, with prior experience in the validation of assessment tools; (3) Dental Surgeon, with a Ph.D. in Oncology, with more than 10 years of clinical experience in oncology and prior experience in the validation of assessment tools; (4) Dental Surgeon, Specialist in Oncology, with 5 years of clinical experience; (5), a professional translator, with a degree in Portuguese Language and Literature, with prior experience in the validation of assessment tools.

Regarding the CVI, all items showed adequate results (CVI ≥ 0.8). Of all the assessed items, six language parameters in four of the scale items, had a score of 0.80; the remainder were equal to 1.0. Table 1 shows the four items that obtained CVI = 0.80.

**Table 1:** Items of the scale that had CVI scores = 0.80.

Item	Phase	Text	CVI		
			Semantics/idiomatic	Cultural	Conceptual
	Original	Please circle one (1) number per line which best indicates how well this statement applied to you during the past 7 days.	–	–	–
	Translation	*Por favor, circule um (1) número por linha que melhor indica quão bem a afirmação se aplicou a você durante os últimos 7 dias.*	–	–	–
	Committee result	*Por favor, circule um (1) número por linha que melhor indica quão bem a afirmação se aplicou a você durante os últimos 7 dias.*	1.0	0.8	1.0
SSS-7	Original	I enjoy going out in public.	–	–	–
	Translation	*Eu gosto de sair em público.*	–	–	–
	Committee result	*Eu gosto de sair na rua*	1.0	0.8	1.0
SSS-14	Original	I sense that others feel strained when around me.	–	–	–
	Translation	*Eu sinto que os outros se sentem tensos quando estão perto de mim*	–	–	–
	Committee result	*Eu sinto que os outros se sentem tensos quando estão perto de mim*	1.0	0.8	1.0
SSS-17	Original	I feel sorry about things I have done in the past	–	–	–
	Translation	*Eu me arrependo de coisas que fiz no passado*	–	–	–
	Committee result	*Eu me arrependo de coisas que fiz no passado*	0.8	0.8	0.8

All other items of the scale showed CVI = 1.0 (for all assessed criteria).

One of the study authors, who originally developed the scale, evaluated the back-translated versions and considered the process appropriate. Moreover, he was available to help throughout the entire translation process.

### Pretest

For the pretest, 15 patients diagnosed with HNC after appearance-altering surgical treatment and who were treated at the Dentistry Department of an oncological hospital for creation and/or maintenance of a maxillofacial prosthesis and palate obturator prosthesis were included. No patient refused to participate in the study.

The mean age of patients was 63.2 years (SD = 15.37); 8 (53.3%) were males; 12 (80.1%) patients had low family income and 9 (60%) had not finished elementary school, i.e., had low level of schooling.

Patients were given the option to either self-administer the tool or to have it applied by the interviewer. Only 1 (6.7%) chose to self-administer it. In all other interviews, the SSS was applied by the interviewer.

All patients answered a questionnaire with questions related to the understanding of each SSS item and their respective answers. Table 2 shows the results about the scale understanding and the changes suggested by the patients interviewed in the pretest. The only suggestion for change (replacing the word “cancer” by “treatment”) (Table 2) was sent to the committee of experts who decided there was no need to make the change, keeping the items in the original form.

**Table 2:** Results of the SSS understanding by patients interviewed during the pre-test phase.

Variable	Category	*n*	%
*Patient assessment*			
Patient understood the entire scale	Yes	13	86.6
	No	2	13.4
Item that patient did not understand	SSS-14	1	6.7
Suggestion for change by the patient	Yes	1	6.7
	No	14	93.3
Suggested change	Replace the word “Cancer” by “Treatment”	1	6.7

*Researcher's assessment*			
Patient understood the entire scale	Yes	14	93.3
	No	1	6.7
Item that patient did not understand	Understanding of the Likert-type scale	1	6.7

From the translation process to the pre-test, the SSS remained similar to the original scale. Table 3 shows the translated and culturally adapted version of scale in the Portuguese language (Brazil).

**Table 3:** Shame and Stigma Scale (SSS) in Portuguese (Brazil), final version - 20 items. Find below a list of statements. Please circle one (1) number per line which best indicates how well this statement applied to you during the past 7 days.

	Nunca	Raramente	Às vezes	Frequentemente	O Tempo todo
1. Eu gosto da minha aparência	0	1	2	3	4
2. Eu evito me olhar no espelho	0	1	2	3	4
3. Eu tenho vergonha da minha aparência	0	1	2	3	4
4. Eu estou feliz com a aparência do meu rosto ou do meu pescoço	0	1	2	3	4
5. Eu sinto que as pessoas ficam me encarando	0	1	2	3	4
6. Eu evito encontrar pessoas por causa da minha aparência	0	1	2	3	4
7. Eu gosto de sair na rua	0	1	2	3	4
8. Eu me sinto angustiado pelas mudanças em meu rosto ou pescoço	0	1	2	3	4
9. Eu sinto que os outros me consideram responsável pelo meu câncer	0	1	2	3	4
10. Eu sinto vergonha quando eu conto meu diagnóstico para as pessoas	0	1	2	3	4
11. Eu sinto vergonha por ter desenvolvido o câncer	0	1	2	3	4
12. As pessoas me evitam por causa do meu câncer	0	1	2	3	4
13. Eu sinto vontade de manter meu câncer em segredo	0	1	2	3	4
14. Eu sinto que os outros se sentem tensos quando estão perto de mim	0	1	2	3	4
15. Eu tenho um forte sentimento de arrependimento	0	1	2	3	4
16. Eu faria muitas coisas diferentes se eu tivesse uma segunda chance	0	1	2	3	4
17. Eu me arrependo de coisas que fiz no passado	0	1	2	3	4
18. Eu sinto vergonha da mudança em minha voz	0	1	2	3	4
19. Eu evito falar com as outras pessoas	0	1	2	3	4
20. Eu consigo participar de conversas	0	1	2	3	4

## Discussion

The present study carried out the translation and cultural adaptation into Portuguese (Brazil) of the SSS. Throughout all the study phases, we used a consolidated systematic methodology and internationally accepted norm for the effectiveness of the process of translation and cultural adaptation of health assessment tools. This step is one of the most important in the validation process and must be very carefully performed so that future studies to evaluate the psychometric properties can confirm the scale validity.^13^, ^16^

Ideally, we seek to validate existing tools, so that the development of a new one is not necessary. The validation of an existing tool helps to use the same data pattern in different populations, when carrying out further studies and international comparisons. This process also saves time and money, when compared to the complex process of developing new tools.^14^

After the translations are performed, it is important that the tool be sent to the committee of experts, so the first phase of cultural adaptation can be carried out, before it is submitted to the pretest. This step, which has been sometimes neglected, assists in the way the patient understands and answers the tool questions.^13^, ^16^

As expected, the Brazilian population still has problems regarding education. Therefore, considering that the hospital where the study was performed is a reference center in public oncological care in Brazil, we identified a sample in the pretest with low level of education. In a study performed in São Paulo, from 2000 to 2006, more than 40% of patients with HNC were illiterate or had not finished Elementary School.^17^ For this reason, it is important for assessment scales to be concise and well-understood by responders.

The SSS has 20 items comprising short statements, which can be self-applied or applied by the interviewer. All patients were asked about their preferences for the scale response method (applied by the interviewer or self-applied). Although the original scale was developed to be self-applied, only one patient chose to do it so, whereas all the other 14 chose to have the scale applied by the interviewer. Although this may be considered a study limitation, the authors believe that the inclusion only of patients able to appropriately answer the scale when it was self-applied would not represent the population being studied (which often has low level of schooling). Interestingly, a previous Brazilian study found that 77% of patients prefer that health assessment tools be applied by an interviewer.^18^

This study was prompted by the scarcity of tools to assess body self-image of patients with HNC, and especially tools already validated for the Portuguese language. Tools such as the Quality of life - Head and Neck Cancer Module EORTC H&N35, FACT-HN and University of Washington UWQOL are available for use in the Portuguese language, but none of them include specific items related to shame and/or stigma.

## Conclusion

The Portuguese version of the SSS was considered adequate and culturally adapted for use in Brazil. Therefore, the tool can be applied to the assessment of its psychometric properties in the future.

## Conflicts of interest

The authors declare no conflicts of interest.
